# Anticancer Activities of Natural and Synthetic Steroids: A Review

**DOI:** 10.1021/acsomega.4c08577

**Published:** 2025-02-19

**Authors:** Daniel F. Mendoza Lara, M. Elena Hernández-Caballero, Joel L. Terán, Jesús Sandoval Ramírez, Alan Carrasco-Carballo

**Affiliations:** † Laboratorio de Elucidación y Síntesis en Química Orgánica, ICUAP, BUAP, Puebla, Pue, Mexico City, México 03940; ‡ Facultad de Medicina, BUAP, Puebla, Pue, Mexico City, México 03940; § Centro de Química, ICUAP, BUAP, Puebla, Pue, Mexico City, México 03940; ∥ Laboratorio de Síntesis y Modificación en Productos Naturales, FCQ, BUAP, Puebla, Pue, Mexico City, México 03940; ⊥ CONAHCYT, LESQO, ICUAP, BUAP, Puebla, Pue, Mexico City, México 03940

## Abstract

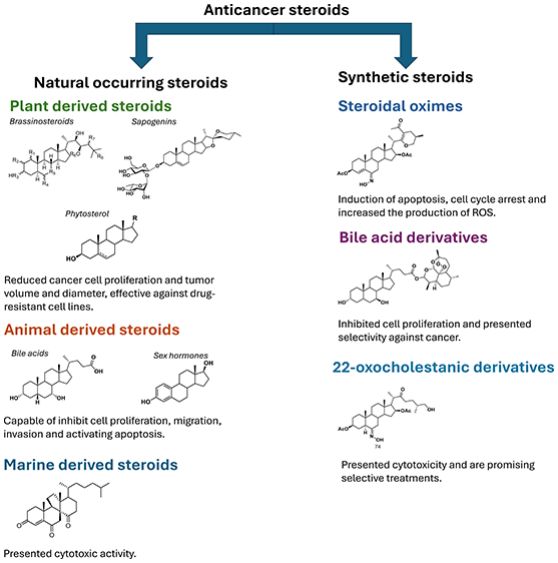

Steroids have demonstrated a wide field of research on the subject of anticancer compounds, particularly antiproliferative with cell lines, with special emphasis on the historical link between steroids and cancer and the use of in silico technologies to understand the impact of natural and synthetic steroids on cancer cells focused on finding common denominators of the type of structural changes that give antiproliferative and/or cytotoxic properties, both in control and cancer cell lines. Through this review and classification by origin and/or synthesis, it is found that steroidal saponins are highly cytotoxic, although with low selectivity against control cells, while on the part of the aglycone the presence of heteroatoms such as nitrogen and oxygen increases the antiproliferative activity, mainly via cell cycle arrest and the induction of apoptosis, mechanisms that have been partially proven, using semisynthetic derivatives, as well as bioconjugates between saponins and nitrogenous steroids with now a high cytotoxicity and selectivity against control cell lines. This gives rise to the idea that steroids as a study model for the design of anticancer agents are an excellent template with a wide field of study.

## 1 Introduction

**Figure 1:**
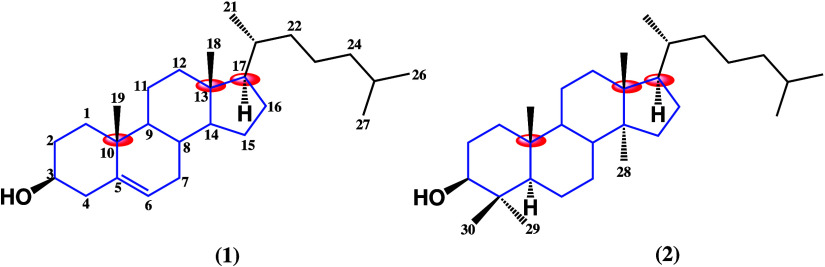
Cholesterol (steroid) (**1**) and lanosterol (triterpene) (**2**).

Steroids are one of the largest families of natural products synthesized by eukaryotic cells. Steroids contain a common structure composed of four cycloalkane fused rings as seen in Figure 1 (cholesterol (**1**) as an example). Usually, this nucleus presents methyl groups at C-10 and C-13 positions and an aliphatic substituent at C-17 called a “side chain”, which confers on them a broad range of chemical diversity. Natural steroids play important roles in all living beings; in mammals, their biosynthesis starts from acetyl coenzyme a via the triterpene lanosterol (**2**). This last step undergoes enzymatic chemical modifications and loses three carbon atoms (C-28, C-29, and C-30) to generate cholesterol.^1,2^

**Figure 2:**
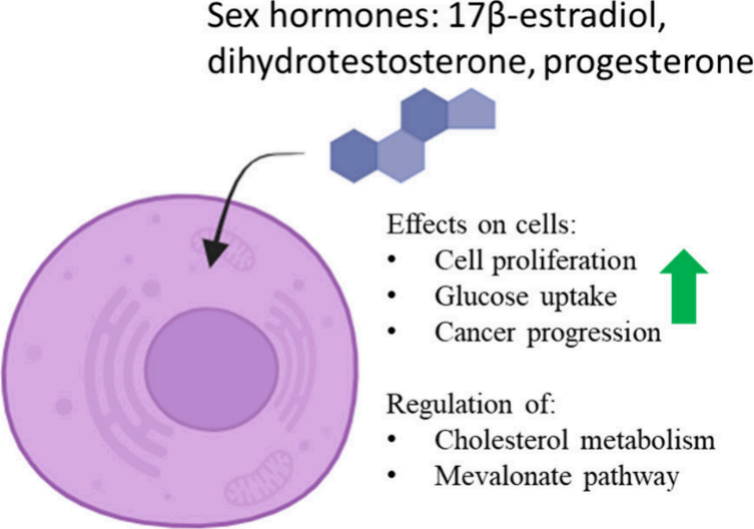
Molecular mechanism of steroids’ effect on cells. Built from refs (12−16.)

Due to their structural diversity, steroids have been classified into different groups according to their source or the chemical moiety contained in their structure. Steroids are synthesized in terrestrial and aquatic living organisms, such as plants, fungi, algae, yeasts, animals, and humans. The presence of steroids on earth has been found in Mesozoic fossils, revealing the importance of these compounds in life.^3,4^ The relationship between cancer and steroids has been studied for a long time, revealing that animal and human castration influences the development and function of multiple cell types and organs such as prostate and mammary glands.^5,6^ Ch. B. Huggins was a pioneer in establishing the relationship between symptomatic prostate cancer progression and human sex steroids, reporting that androgen deprivation, via castration, can be used as a treatment for prostate cancer and metastatic castration-sensitive prostate cancer.^7,8^ Nowadays, *in silico* technologies allow for a better understanding of the effect of steroids on cancer cells, as depicted in Figure 2. Sex steroids like testosterone and 17β-estradiol are capable of stimulating cell proliferation and their differentiation and regulating cell metabolism, acting through androgen and estrogen receptors localized in the cytoplasm. Once bound to their respective receptor, they dimerize cells and translocate to the nucleus where they act as regulators, promoting or inhibiting the expression of genes related to cell proliferation.^9−11^ This important mechanism could occur in many types of cancers, but it has been observed, especially in those falling into the “hormone-dependent cancers” category. The latter are those that arise from sexual organs or mammary tissues, such as ovarian, breast, prostate, and endometrial cancer and others.^12−14^

Due to this direct interaction with cancer development, a search for new steroidal structures that can act as anticancer agents has been launched, mainly from those having structural similarity to sex hormones.^12^ The isolation and evaluation of novel steroids from natural sources have been reported in the literature, and at the same time, there are investigations centered on chemical modifications of known steroids to enhance their anticancer activity.^13−15^ A review of articles ranging from 2020 to 2023 is presented along with previous specific articles to put steroids as anticancer agents into context, highlighting the anticancer activity of natural and synthetic steroids. This review is divided into plant-derived steroids, animal-derived steroids, and synthetic steroids depending on the steroid origin.

## 2 Plant-Derived Steroids

Plants are one of the major natural sources of steroidal structures; their metabolic pathways allow them to synthesize structures that are not found in other kingdoms. In addition, these structures show interesting bioactivity that is used in many fields, such as medicine, veterinary medicine, agriculture, the cosmetic industry, and others.^15−17^ Due to this, many studies have emerged, pointing out their potential application against cancer.

### 2.1 Steroidal Sapogenins

**Figure 3:**
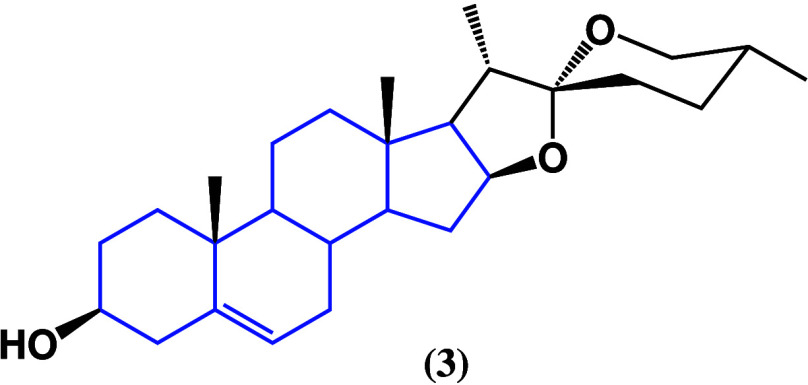
Structure of diosgenin (**3**).

Diosgenin (**3**), shown in Figure 3, is a steroidal sapogenin derived from plant sources such as *Dioscorea composita*. **3** has demonstrated important bioactivity against a diverse panel of cancers including lung cancer, cervical cancer, prostate cancer, glioma, and leukemia.^18^

In breast cancer models, some studies have reported that diosgenin influences several signaling pathways crucial to the progression of breast cancer, including FoxO, PI3K-Akt, p53, Ras, and MAPK signaling. This could potentially result in the deceleration of cell growth, contrary to the Warburg phenomenon, and the induction of cytotoxic effects on breast cancer cells.^19^

### 2.2 Brassinosteroids

**Figure 4:**
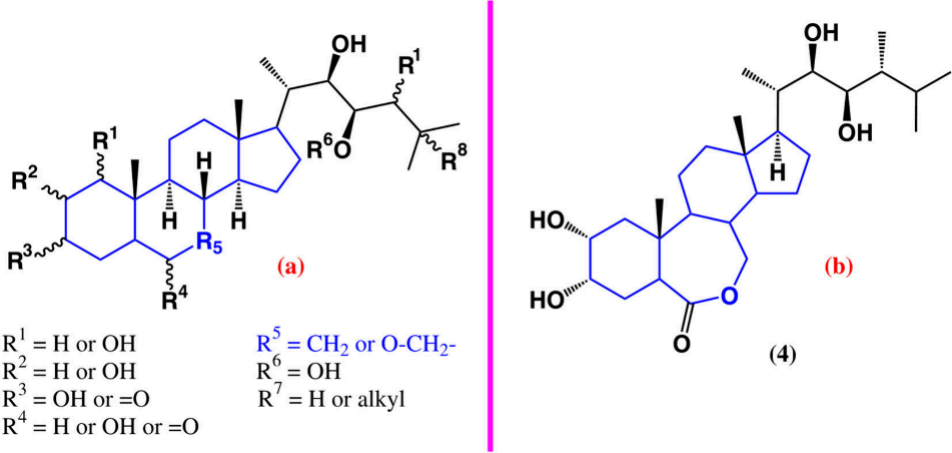
(a) General structure of brassinosteroids. (b) Structure of epibrassinolide.

Brassinosteroids are a unique class of secondary metabolites that possess the strongest plant growth activity. They have been related to vital processes, such as seed germination and cell division.^20,21^ Due to these last-mentioned activities, they have been successfully tested as anticancer agents. The structures of brassinosteroids bear hydroxyl and ketone at ring A, a ketone or a lactone at ring B, and hydroxyl groups at the side chain, with diverse stereochemistry at hydroxyl and methyl groups. Their general chemical structure is shown in Figure 4A.

It has been reported that brassinosteroids decrease cell proliferation and induce apoptosis in colon cancer cell lines; e.g. the administration of epibrassinolide (**4**) (Figure 4B) reduced the volume and diameter and caused cell cycle arrest of colon tumor cells in mouse xenograft models when applied in a dose-dependent manner.^22^ In neuroblastomas, it was able to induce apoptosis by interfering with the phosphorylation of GSK3β and preventing the translocation of β-catenin.^23^

Epibrassinolide, in combination with gemcitabine, had a synergetic effect on pancreatic cancer cells, inducing apoptosis via estrogen receptors and reducing the epithelial–mesenchymal transition, a key step in the metastasis process.^24^

**Figure 5:**
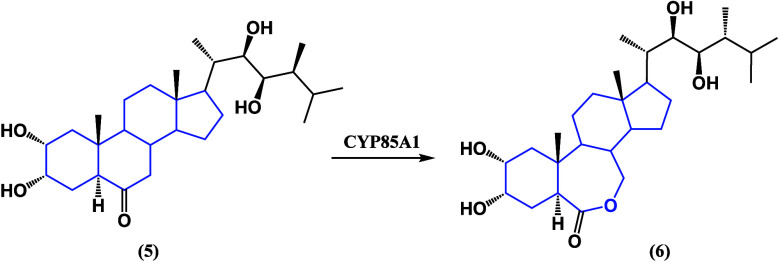
Last step of the biosynthesis of brassinolide (**5**) from catasterone (**6**).

Castasterone (**5**) and brassinolide (**6**) have been evaluated against cancer. The first is an intermediary metabolite in **4** biosynthesis (Figure 5).^25^ Its anticancer activity has been evaluated *in vitro* against small-cell lung cancer cell lines H69 and VPA17, finding that **6** is cytotoxic for both cell lines with an IC_50_ value of 1.0 μM. The incubation of the drug-resistant cell line VPA17 with **6** for 96 h made them sensitive to etoposide and doxorubicin.^26^

### 2.3 Steroidal Saponins

Steroidal saponins are natural secondary metabolites widely distributed in plants, possessing various structures and functions. Their structures are complex and composed of a carbohydrate moiety and a hydrophobic structure known as aglycone which can be steroid- or triterpenoid-based.^27,28^ They are reported to have a wide range of properties; for example, they can act as defensive molecules against insects and pathogens but also have interesting pharmacological activity.^29−31^

**Figure 6:**
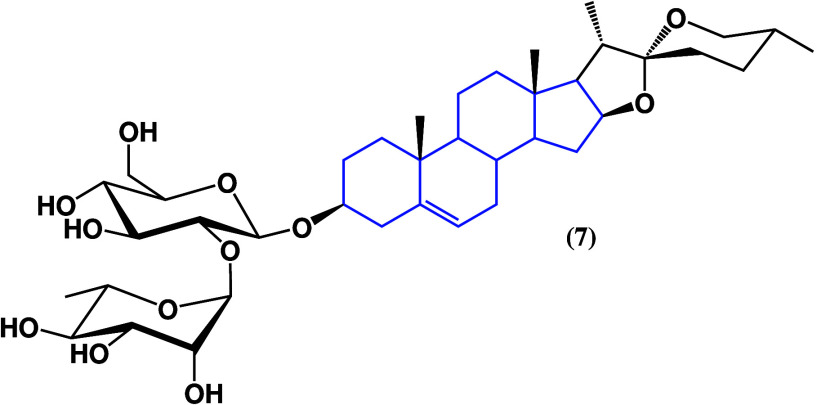
Progenin III structure (**7**).

Among all saponins, spirostan saponins have proven to be some of the most bioactive compounds against cancer; progenin III (**7**) (Figure 6)^32^ exhibited extensive activity on a broad range of cell lines, including sensitive and drug-resistant phenotypes. Table 1 shows the recorded IC_50_ value for some drug-resistant cell lines, showing an effect on breast, colon, liver, and glioblastoma cancer cell lines as well as human T lymphoblasts and hepatocellular carcinoma, indicating a high anticancer potential but low selectivity against noncancerous cells and the need for modifications of the structure to regulate such activity.

**Table 1:** Bioactivity of Progenin III (**7**) against Cancer Cell Lines

Cell line	IC_50_ value (μM)
CCRF-CEM	1.59 ± 0.31
CEM/ADR5000	1.70 ± 0.66
MDA-MB-231-PCDNA	3.17 ± 0.42
MDA-MB-231-BCRP	4.22 ± 0.13
HCT116 P53^+/+^	3.43 ± 0.29
HCT116 P53^–/–^	3.69 ± 0.40
U87MG	3.13 ± 0.17
U87MGΔEGFR	4.77 ± 0.36
HEPG2	10.24 ± 0.71
AML12	23.82 ± 1.95

**Figure 7:**
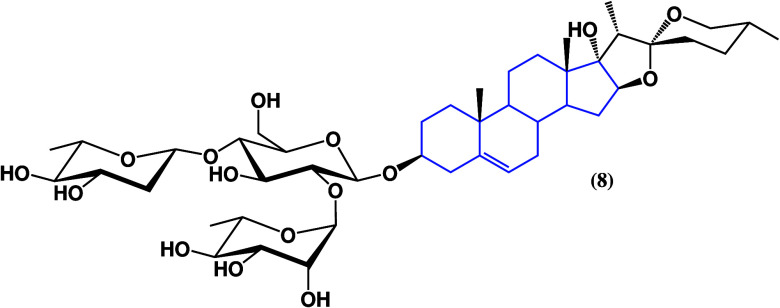
Structure of PP9 (**8**).

Another report shows the evaluation of the spirostan saponin PP9 (**8**)^33^ as an anticancer agent (Figure 7), a compound isolated for the first time from the rhizomes of *Paris polyphylla*. Results indicate that PP9 does not affect normal colorectal cells in an NCM460 cell culture, showing selectivity toward cancer. However, it does inhibit, in a dose-dependent manner, the proliferation of colorectal cancer cell cultures HT-29 and HCT116. Furthermore, PP9 effectively induced G2/M phase arrest by upregulating p21, suppressing cdc25C, cyclin B1, and cdc2, and stimulating cell apoptosis, showing as **8** if it presents selectivity toward tumor cells versus healthy cells.

**Figure 8:**
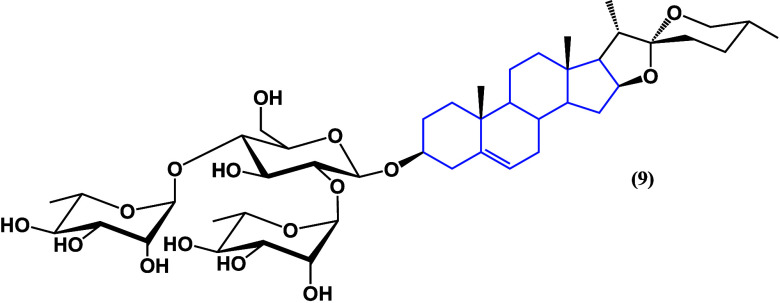
Structure of dioscin (**9**).

Despite its recognized efficacy, the specific mechanisms through which dioscin (**9**) (Figure 8) acts against prostate cancer remain unclear. It has been demonstrated that dioscin (**9**) may inhibit cell growth and invasion by increasing SHP1 phosphorylation [p-SHP1 (Y536)] and subsequently inhibiting the P38 mitogen-activated protein kinase signaling pathway, suggesting its potential as a therapeutic option for both androgen-sensitive and androgen-independent prostate cancer.^34^

Dioscin has also been proven effective against oral squamous cell carcinoma cells by diminishing surviving levels and disrupting EGFR binding. *In vivo* studies confirm dioscin’s efficacy in suppressing tumor development, highlighting its potential as a promising treatment strategy through targeting the EGFR-surviving axis,^35^ resulting in a particular focus on EGFR as a target for the search for new anticancer steroidal saponins.

**Figure 9:**
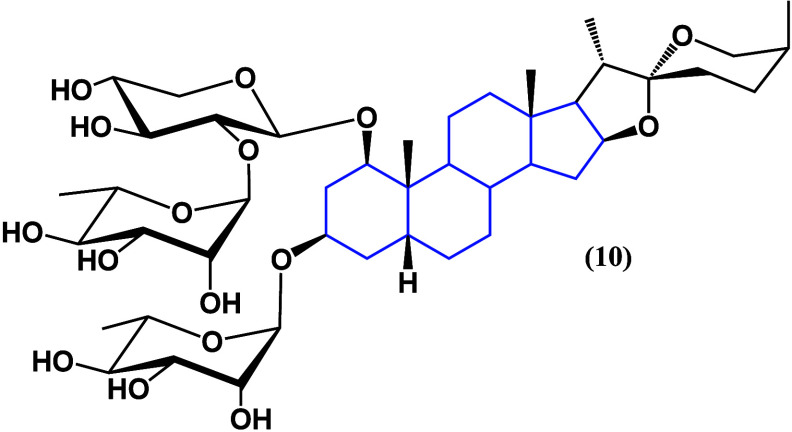
Structure of T-17 (**10**).

In gastric cancer, the spirostan saponin T-17 (**10**) (Figure 9) shows dose-dependent cytotoxicity in cell lines SGC-7901 and AGS. Also, it induces apoptosis via caspase activation as well as G0/G1 cell arrest. According to Western blot analysis, the expression of Beclin-1 was increased while that of p62 was decreased, indicating the promotion of autophagy.^36^

**Figure 10:**
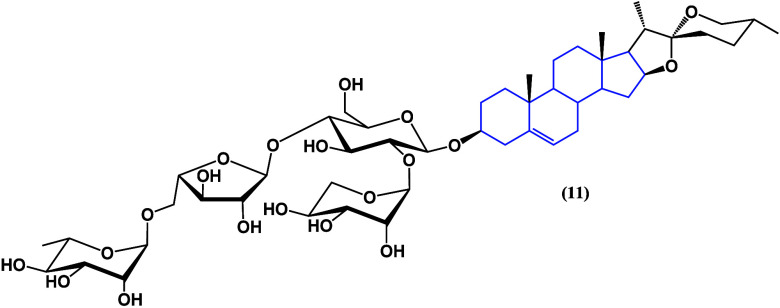
Isolated structure from *Aspidistra triradiate* (**11**).

The extraction of *Aspidistra triradiata* provided the (22S,25R)-spirost-5-ene-3β-yl-O-α-l-rhamnopyranosyl-(1 → 2)-O-[O-α-l-rhamnopyranosyl-(1 → 5)-α-l-arabinofuranosyl-(1 → 4)]-β-d-glucopyranoside (Figure 10),^37^ a new tetrasaccharide spirostan saponin which was characterized by NMR spectroscopy and mass spectrometry. Compound **9** showed strong cytotoxic activity against SKU-LU-1, HT-29, MCF7, and HepG2 cancer cell lines with IC_50_ values ranging from 0.28 to 0.81 μM, giving a selective effect on cancer cell lines mainly through cytotoxic pathways, although its mechanism of action is still unknown.

**Figure 11:**
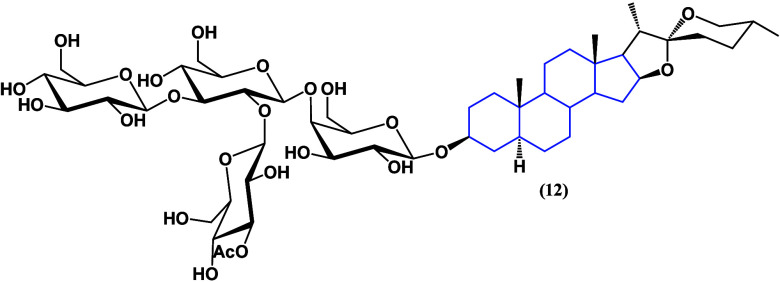
Compound A-24 (**12**).

A-24 (Figure 11) demonstrates dose-dependent cytotoxicity in gastric cancer cell lines (SGC-7901 and AGS), inducing apoptosis, autophagy, G2/M phase arrest, and the modulation of cell cycle-related proteins. The compound downregulates the PI3K/Akt/mTOR pathway and its downstream targets,^38^ emphasizing cell cycle arrest pathways as the main mechanism at low doses, which indicates that this type of saponin is promising for future studies given the potential to be selective against carcinogenic cells.

Steroidal saponins show delayed activity depending on the coupled carbohydrates as well as on the steroid, showing that diosgenin derivatives with 3 carbohydrate units present greater selectivity toward cancer cells, while smilagenin and sarsapogenin derivatives affect healthy and cancer cells indistinctly.

### 2.4 Phytosterols

**Figure 12:**
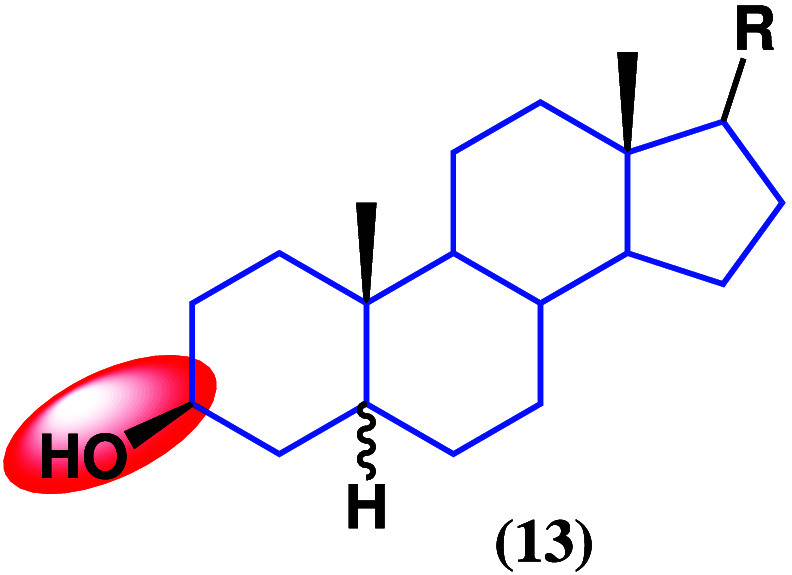
Structure of phytosterols (**13**).

Phytosterols are natural plant-derived compounds which have proven to be beneficial in humans by reducing serum cholesterol agents^39,40^ and have anti-inflammatory^41,42^ and antitumor effects, among others. Their basic structure (**13**) is shown in Figure 12, bearing hydroxyl groups in diverse positions as well as double bonds.^43^ These compounds are present in most plant species consumed by humans, and their intake has been related to the reduction of digestive system-related cancer incidence.^44,45^

**Figure 13:**
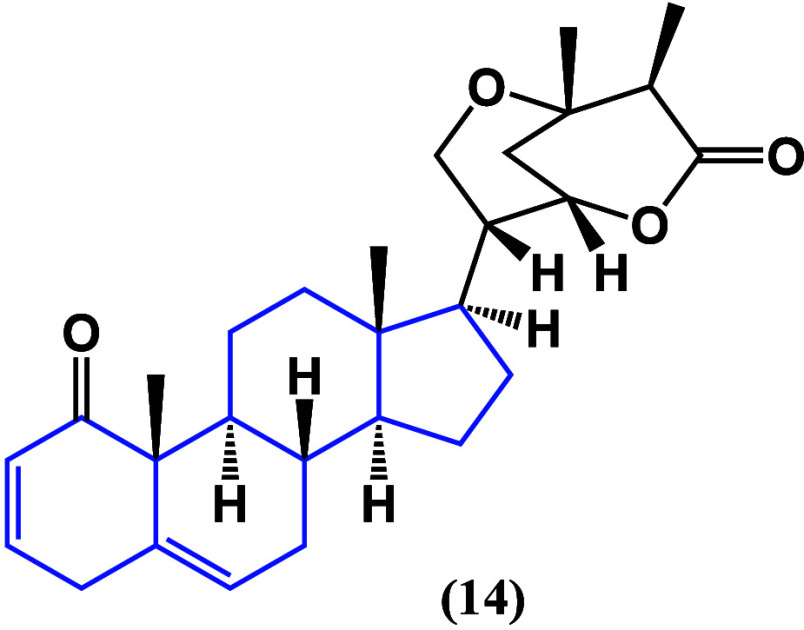
Structure of phytosterol RinoxiaB (**14**).

RinoxiaB (Figure 13), a novel phytosterol isolated from *Datura innoxia*,^46^ was tested against human colon adenocarcinoma cell line HCT 15, demonstrating it to have antiproliferative activity with an IC_50_ value of 4 μM. Also, it was proven by flow cytometry analysis, that the cell cycle arrest was in phases S and G2/M and Western blot revealed an up-regulate of Bcl-2 protein, indicating an induction of apoptosis due to mitochondrial damage and subsequent cytochrome C release.^47^

**Figure 14:**
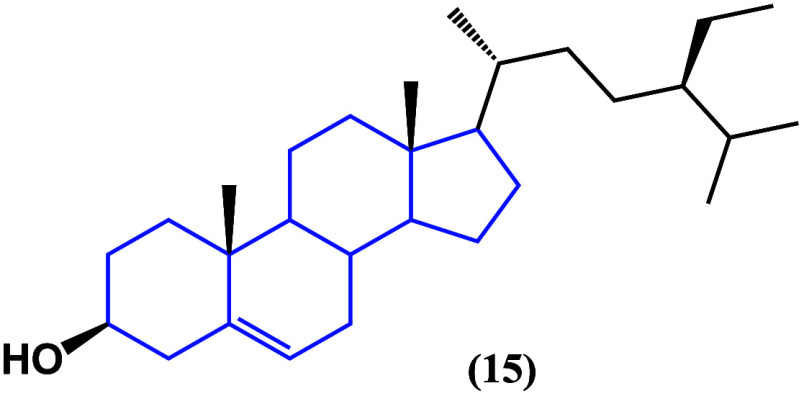
Structure of β-sitosterol (**15**).

A different type of digestive system cancer was studied.^48^ Employing colorectal cancer, it was found that β-sitosterol (**15**) (Figure 14) can recover oxaliplatin sensitivity in drug-resistant colorectal cancer by inhibiting the expression of BCRP. This study also demonstrated that the p53 pathway is also affected, being capable of disrupting the p53-MDM2 interaction and causing the inhibition of the NF-κB factor.

**Figure 15:**
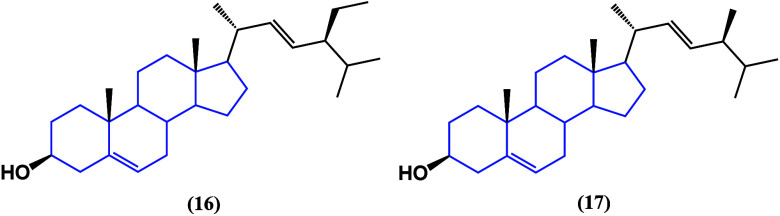
Structures of stigmasterol (**16**) and brassicasterol (**17**).

β-Sitosterol (Figure 15) and two other sterols—stigmasterol (**16**) and brassicasterol (**17**), isolated from *Rhizophora apiculata*—have been studied against HeLa, MCF-7, and A549 cancer cell lines (Table 2).^48^ The biological evaluation showed that those structures having C-22 and C-24 side chains and 24-methyl and 24-ethylsterol moieties increased the cytotoxicity. Biological evaluation showed that those structures with C-22 and C-24 side chains, 24-methyl and 24-ethylsterol fractions, increased cytotoxicity, specifically in hormone-dependent cell lines of breast and cervical cancer, although the absence of carbohydrates generates a decrease in the potency of the activity, although a latent cytotoxic effect is present.

**Table 2:** Cytotoxic Activity of Selected Compounds

	IC_50_ value (μM)		
**Steroids**	**MCF-7**	**A549**	**HeLa**
β-Sitosterol	125.29 ± 2.1	114.04 ± 2.4	88.75 ± 4.7
Stigmasterol	67.95 ± 1.5	54.9 ± 2.6	71.2 ± 2.5
Brassicasterol	78.88 ± 1.3	61.77 ± 1.8	79.04 ± 1.4

**Figure 16:**
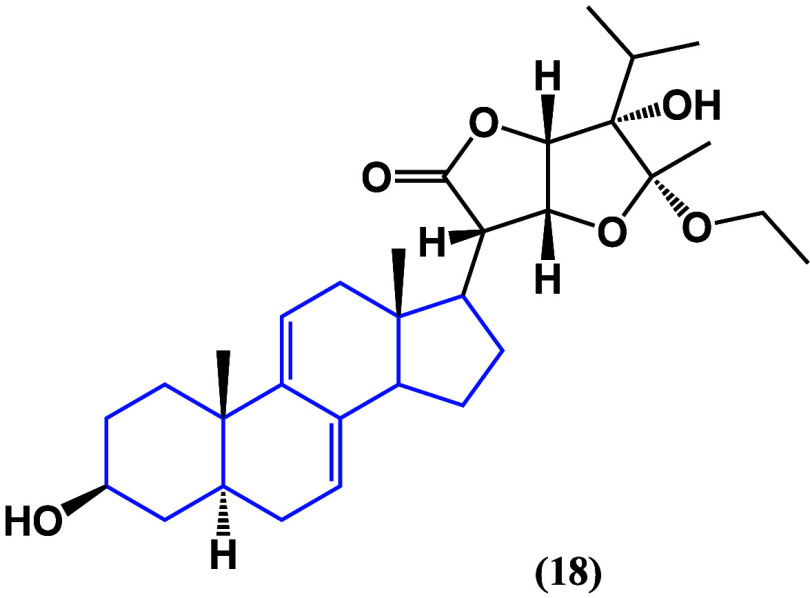
Structure of a stigmastadiene phytosterol (**18**).

Stigmasterol has proven to be effective against glioblastoma in combination with other phytochemicals, such as isoflavones and xanthones. The chloroform fraction of *Moraea sisyrinchium* was evaluated,^49^ reporting the inhibition of proliferation and migration by inducing cycle arrest and apoptosis. Phytosterols have proven to be effective against female hormone-related cancers, such as cervical, ovarian, and endometrial cancer. For the latter, stigmasterol was found to have an antiproliferative effect, and it was associated with G1 cell cycle arrest. Cell migration is a well-known key process in metastasis; stigmasterol also inhibited this process as well as tumorspheres by inhibiting SLUG, SNAI1, and β-catenin.^50^ The stigmastadiene phytosterol (**18**), isolated from *Vernonia amygdalina* (Figure 16),^51^ had remarkable anticancer activity when tested against the HeLa cell line, with an IC_50_ value of 22.44 μM. The molecular mechanism of action indicated that apoptosis is triggered through a caspase pathway. In addition, it could induce cell cycle arrest in the S phase via suppression of P13K/AKT/mTOR.

**Figure 17:**
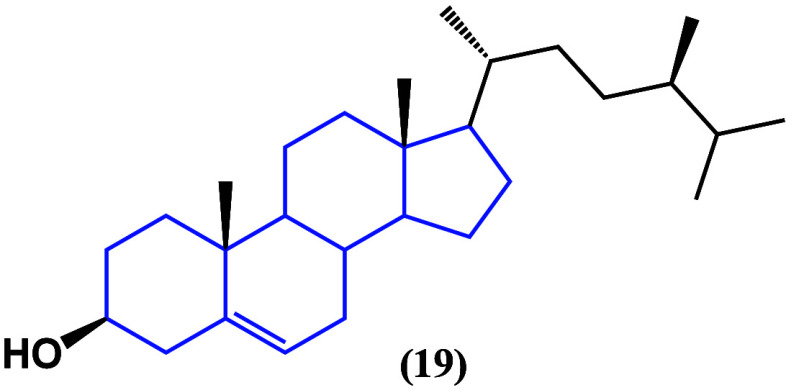
Structure of campesterol (**19**).

The molecular mechanism of the antiproliferative and cytotoxic effect of campesterol (**19**) and β-sitosterol (**15**) (Figure 17) on ovarian cancer was published.^52,53^ The β-sitosterol induced the loss of the mitochondrial membrane potential and increased the generation of reactive oxygen species. On the other hand, campesterol triggered cell death, increased calcium uptake, and enhanced ROS production.

**Figure 18:**
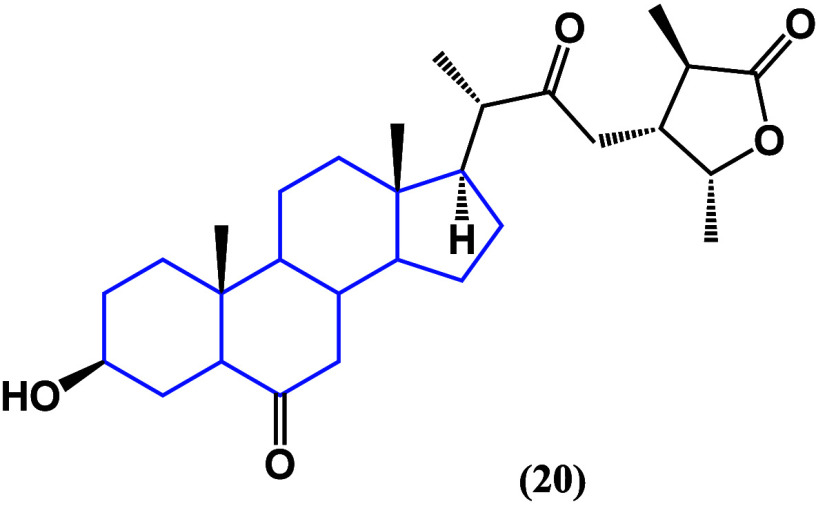
Structure of dendrosterone (**20**).

It is noteworthy to highlight the *in silico* studies that have been performed to propose new possible targets and mechanisms of action of phytosterols. Dendrosterone (**20**) (Figure 18) is a novel stigmastane phytosterol, isolated from *Dendrobium ochreatum*, that has been shown to have a binding energy high enough to be considered a suitable possible ligand against 1M17 protein, which has been related to the progression of multiple types of cancer.^54^

**Figure 19:**
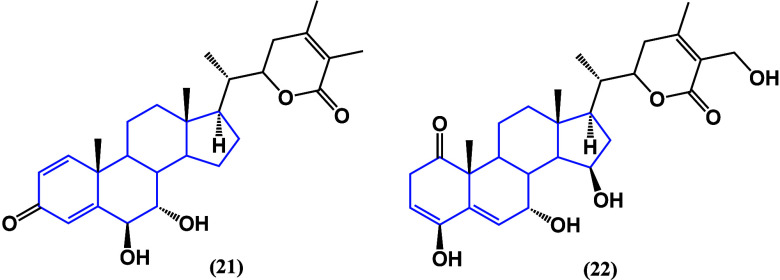
Structure of compounds **21** and **22**.

Compounds **21** and **22** (Figure 19) displayed moderate inhibitory effects on nitric oxide (NO) production in RAW 264.7 cells, exhibiting IC_50_ values of 13.74 and 13.92 μM, respectively. Additionally, they exhibited anti-inflammatory activities by suppressing the production of TNF-α, IL-1β, IL-6, and COX-2. Further insights into the anti-inflammatory mechanism were gained through Western blot analyses, highlighting the inhibition of NF-κB activation.^55^ Promoting antioxidant regulatory factors against cancer cell lines gives rise to the fact that phytosterols are following this route to induce a death mechanism.

### 2.5 Steroidal Alkaloids

**Figure 20:**
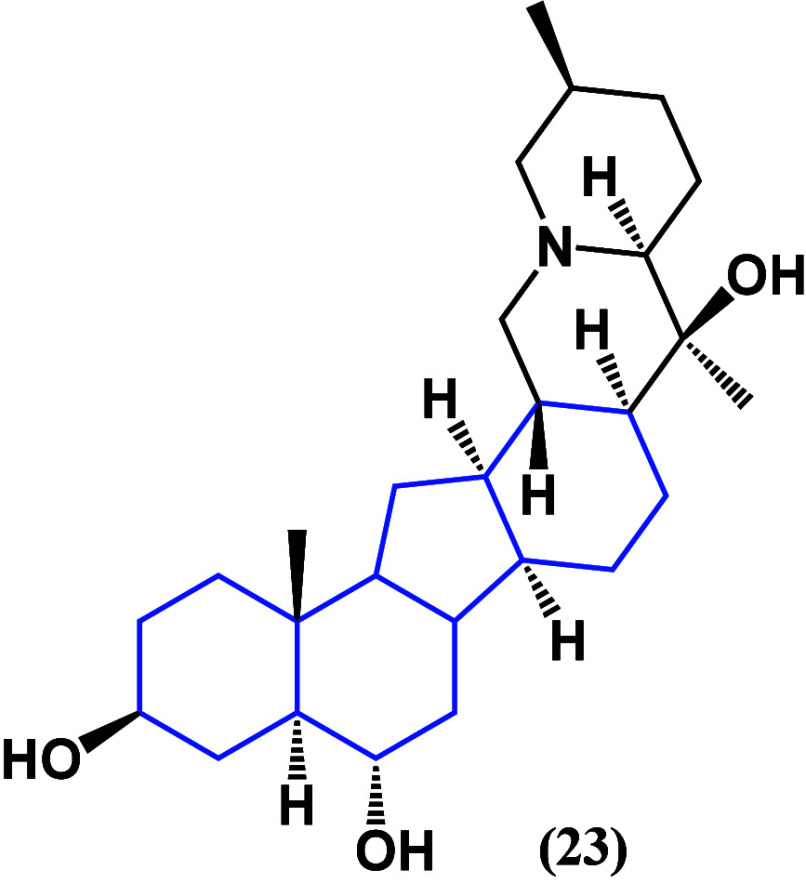
Structure of peimine (**23**).

Steroidal alkaloids are natural nitrogen-containing compounds, synthesized by many organisms but mainly by plants.^56^ A broad range of activities have been related to them, including antimicrobial, anti-inflammatory, and analgesic. An *in silico* screening of plant-based phytosterols identified peimine (**23**) as a lead compound for further research due to its binding affinity with HK2. **23** (Figure 20) has demonstrated cytotoxicity against MRMT-1 breast cancer cells, as evidenced by cell viability assays. Furthermore, its inhibitory effects on HK2 are substantiated by the measurement of intrinsic apoptotic markers, including caspase-9 and cytochrome C. Additionally, the impact on glucose uptake is confirmed through cellular assays, highlighting the multifaceted effects of peimine (**23**) on key pathways associated with cancer cell survival and proliferation.^57^

**Figure 21:**
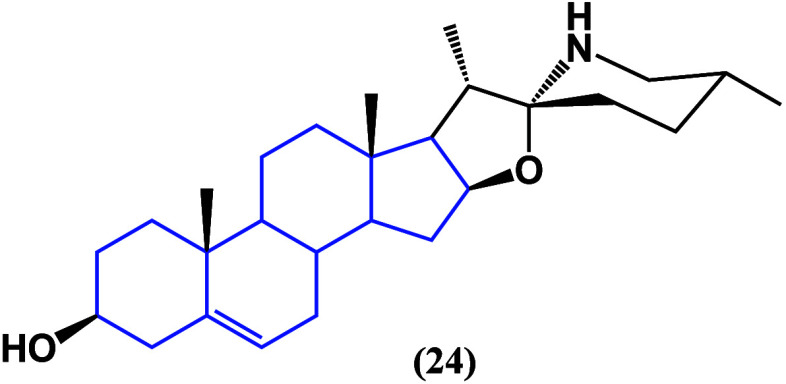
Structure of solasodine (**24**).

Among all steroidal alkaloids, solasodine (**24**) (Figure 21) has been noted to have remarkable activity against cancer. In breast cancer cell lines, MCF-7 decreased the proliferation of tumorspheres while increasing the expression of CD24 by suppression of the Gli1/Hh pathway.^58^ Additionally, studies on bladder cancer revealed that solasodine (**24**) can suppress NRP1 expression and lead to a proapoptotic and antiproliferative effect,^59^ showing a different death pathway than those observed in saponins and phytosterols, particularly because they act at the genetic level by changing CD24 expression to favor apoptotic death processes.

**Figure 22:**
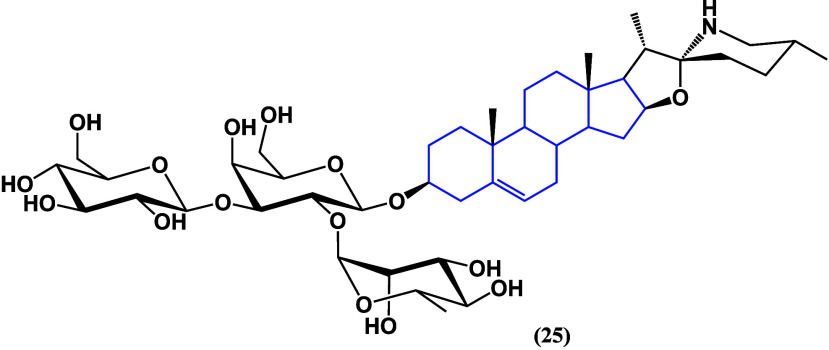
Structure of solasonine (**25**).

Solasonine (**25**) (Figure 22), the saponin of aglycone solasodine, showed interesting activity in PANC-1 and CFPAC-1 pancreatic cell lines, inducing apoptosis while inhibiting their proliferation, migration, and invasion via glutathione metabolism and SLC7A11-mediated ferroptosis. Performing molecular docking studies, it was found that solasonine (**25**) could also interact with TFAP2A.^60^ When combining an azasteroid with a saponin, adding carbohydrates to it, an increase in the antiproliferative biological activity is again observed, combined with the induction of apoptosis.

**Figure 23:**
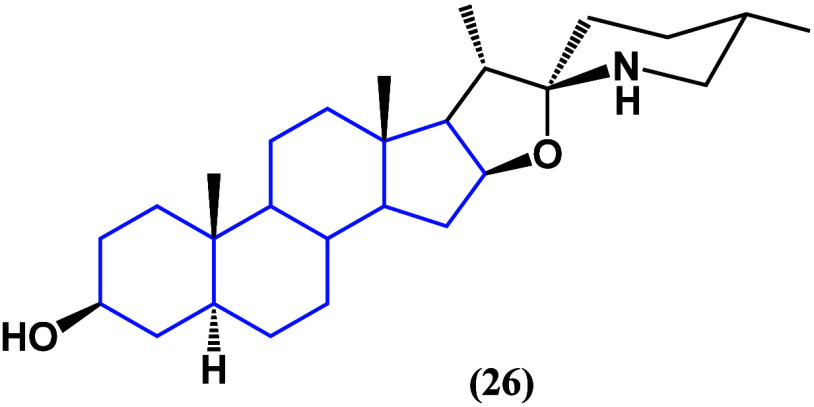
Structure of tomatidine (**26**).

In contrast, in a murine model, tomatidine (**26**) (Figure 23) inhibited gastric cancer tumor growth when administered with a rich tomatidine diet. Also, it inhibited the proliferation of cancer cell culture 85As2. By performing microarrays, it was shown that this diet, rich in tomatidine in the murine model, altered the expression levels of mRNAs belonging to type I interferon.^61^

**Figure 24:**
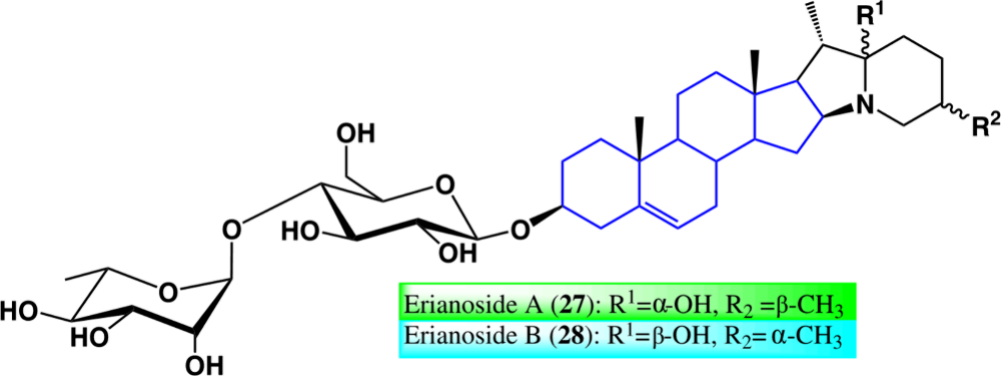
Structures of erianoside A (**27**) and erianoside B (**28**).

Another type of saponins derived from azasteroids, erianosides A (**27**) and B (**28**) (Figure 24), was evaluated against breast cancer, triple-negative, and nontriple-negative cell lines. Erianoside A showed cytotoxicity against T74D with an IC_50_ of 56.39 μM, while erianoside B had no effect. Among some evaluated saponins bearing the rhamnosyl-(1 → 4)-glucose glycone, only erianoside A had moderate cytotoxic activity.^62^ Confirming the combination of carbohydrates with azasteroids, they increase the antiproliferative biological activity

**Figure 25:**
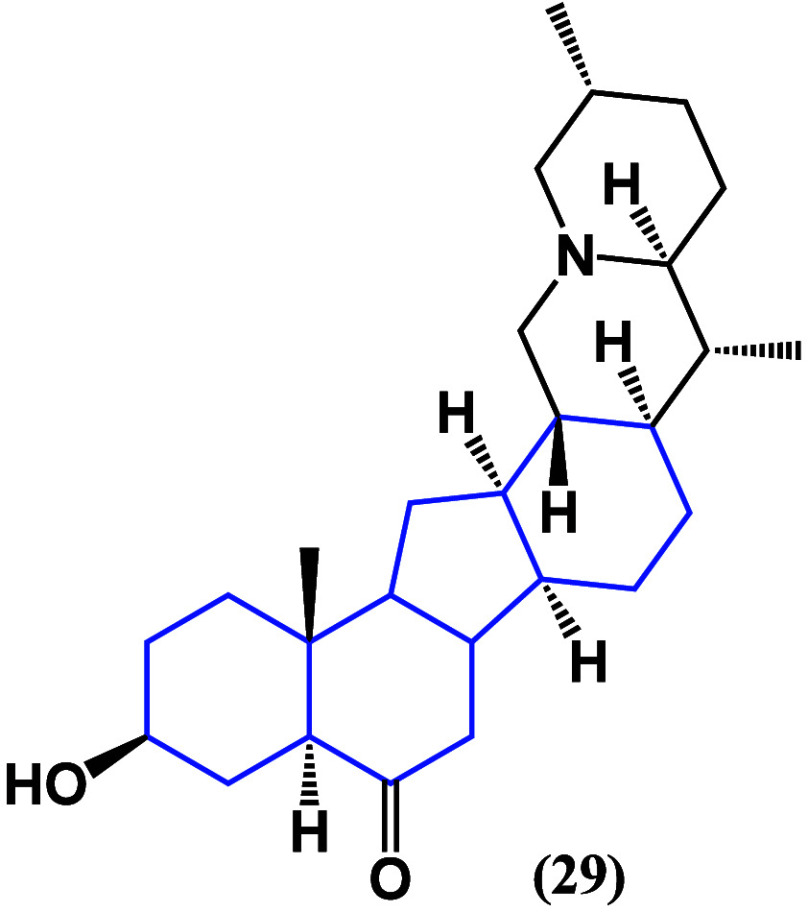
Structure of zhebeirine (**29**).

On the other hand, **29** (Figure 25) showed cytotoxic effects on A549 cell viability with an IC_50_ value of 36.93 μM and inhibited tumor growth in nude mice. These effects are due to a downregulation of CDK1, CDK2, Cyclin A2, and Cyclin B2 and the inhibition of the phosphorylation of p53.^63^ In cell lines associated with lung cancer, it is the aglycone that has the greatest biological potential, focused on the direct or indirect regulation of p53, resuming apoptosis pathways in azasteroidal compounds.

**Figure 26:**
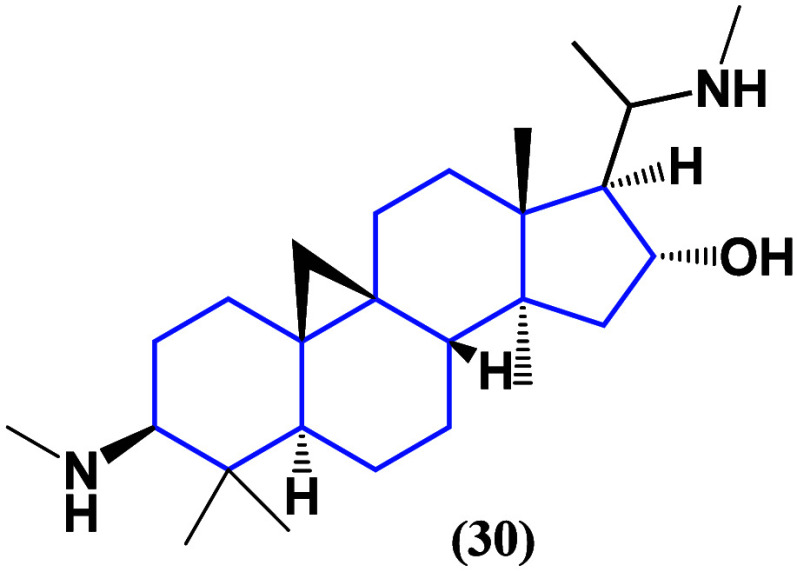
Structure of cyclovirobuxine D (**30**).

Cyclovirobuxine D (**30**) (Figure 26), a hybrid between a steroid and terpenoid, the first due to the basic nucleus as well as the methyls in position 4 characteristic of terpenoids, which is a steroid-like alkaloid, is used in traditional Chinese medicine for the treatment of cardiovascular diseases. Several *in vitro* and *in vivo* studies have shown significant inhibition of non-small-cell lung cancer proliferation, survival, migration, and angiogenesis by inhibiting KIF11-CDK1-CDC25C-cyclin B1 and the κNF-B pathway,^64^ confirming that steroids have direct potential on lung cancer cell lines, focusing on apoptotic pathways

## 3 Animal-Derived Steroids

Steroids from animals have also been tested against multiple types of cancer. In recent years, the evaluation of animal steroids has decreased due to their availability in markets with low yield, high cost, and difficult extraction processes.^65^ These steroids are classified according to their function: sex steroids, bile acids, and corticosteroids.

### 3.1 Bile Acids

**Figure 27:**
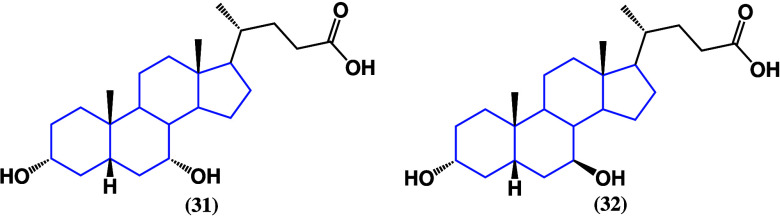
Structures of chenodeoxycholic acid (**31**) and ursodeoxycholic acid (**32**).

Bile acids are a cholesterol-derived family biosynthesized in mammalian livers and secreted alongside cholesterol and phospholipids into the gallbladder.^66^ Human bile is mainly composed of chenodeoxycholic acid with a minor percentage of cholic, desoxycholic, and lithocholic acids. Bile salts act as emulsifying agents to allow the digestion of fat in food.^67^ Despite being related to multiple digestive system diseases, it has been proven that bile acid steroids can have interesting activity against lung cancer and leukemia. Chenodeoxycholic acid (**31**) (Figure 27) was tested against lung adenocarcinoma cell lines, inhibiting cell proliferation, migration, and invasion and activating apoptosis. Western blot and quantitative PCR analysis revealed an increased p53 expression and a decrease in the α5β1/FAK pathway,^68^ reincorporating the trend of p53 activation by cholestanic steroids.

**Figure 28:**
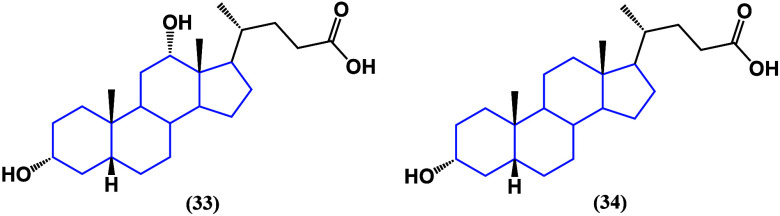
Structures of deoxycholic acid (**33**) and lithocholic acid (**34**).

Chenodeoxycholic acid was also able to suppress acute myeloid leukemia progression in *in vivo* and *in vitro* experiments by causing an excessive production of ROS. This is due to a decrease in the mitochondrial membrane potential and an elevated mitochondrial calcium level. The production of ROS was able to activate p38 MAPK signaling, and chenodeoxycholic acid also caused the inhibition of M2 macrophage polarization.^69^ Ursodeoxycholic acid (**32**) (Figure 27), a bile acid found in bears, has also been tested against cancer. Its action on bile duct cancer cells was evaluated, reporting inhibitory activity of the epithelial–mesenchymal transition by enhancing the E-cadherin expression and suppression of the N-cadherin expression. In glioblastoma, one of the deadliest and most aggressive types of brain cancer, ursodeoxycholic acid induced cell cycle arrest in the G1 phase and subsequent apoptosis. Also, there was an increase in ROS production, and a synergetic effect was observed when evaluated with bortezomib, which has been described as a potential pharmacological agent.^70^ On the other hand, deoxycholic acid (**33**) (Figure 28) has been found to be a promising interfering miR-92b-3p maturation agent, thus acting as a tumor suppressive factor in gallbladder cancer.^71^

Like ursodeoxycholic acid, lithocholic acid (**34**) (Figure 28) has been reported to kill cancer cells in neuroblastomas. Lithocholic acid showed dose- and time-dependent selective effects inducing apoptosis in nephroblastoma cells;^72^ nevertheless, it also showed negative effects on control kidney cells, proving not to be a selective cancer agent. The correlation between serum lithocholic acid levels and the survival of patients suffering gallbladder cancer has been studied, indicating that low levels are related to poor prognoses. Furthermore, treatment of xenografts with lithocholic acid showed a decrease in glutaminase expression, leading cells toward ferroptosis, indicating that it could be used as an antitumor agent.^73^ Nowadays, investigation is focused on the chemical modification of bile acid structures to improve their selectivity and anticancer activity; nevertheless, few new studies have been published recently.

### 3.2 Sex Steroid Hormones and Corticoids

**Figure 29:**
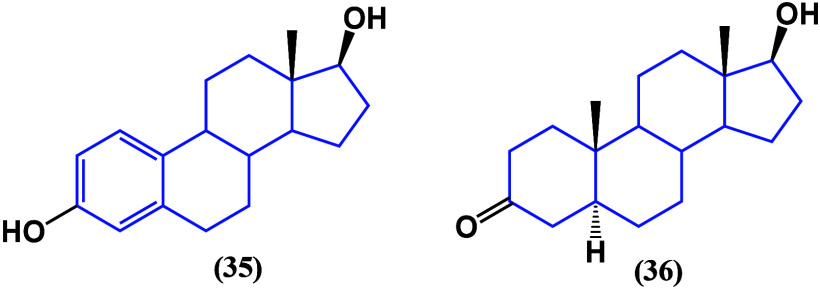
Structures of estradiol (**35**) and dihydrotestosterone (**36**).

Most research on how sex steroids act in cancer cells was conducted during the 1990s, when the discovery and synthesis of sex steroids peaked.^74^ The effect of 17β-estradiol (**35**) and dihydrotestosterone (**36**, Figure 29) was evaluated on tongue cancer cell lines HSC-3 and SCC-25, finding that estradiol, but not dihydrotestosterone, reduced the migration and invasion in both cell lines.^75^

**Figure 30:**
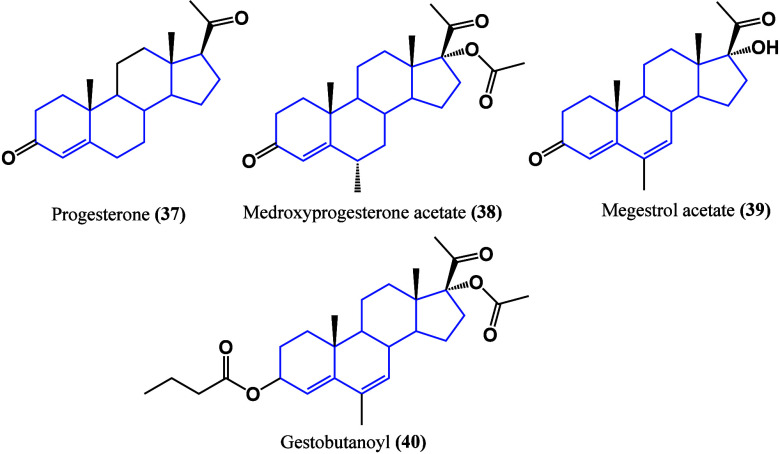
Examples of natural and synthetic progestins.

Another type of sex steroid hormone is progestins (Figure 30), which are well known in the regulation of pregnancy, the menstrual cycle, and cancer proliferation. Recently, they have been proposed as chemosensitizers acting through MDR-related proteins, TGF-β, and Wnt/β-catenin pathways and facilitating apoptosis by disrupting mitochondrial function.^76^ Given its similarity to progesterone **37**, it may directly couple to the progestogen receptor, triggering responses to this pathway in the regulation of cancer cell proliferation.

It has been reported that a combination of 17β-estradiol and progesterone has a synergistic anticancer effect on colorectal cancer in male mice; this effect is possibly related to ERβ, ERα, and PGR-mediated pathways.^77^ Corticosteroids, on the other hand, have recently been related to poor outcomes when coadministered in non-small-cell lung cancer patients under immunotherapy.^78^ The same results were observed^79^ when administering high doses of corticosteroids in melanoma, non-small-cell lung cancer, and renal carcinoma patients under anti-PD1 therapy.

### 3.3 Marine-Derived Steroids

Oceans represent an important source of chemically complex structures such as polyhydroxysteroids, epoxysteroids, and pregnane-type steroids, among others. It is difficult to establish a common structure in marine-derived steroids.^80,81^ The three main sources of marine steroids are sponges, corals, and sea stars; this review article deals with these groups according to their biological activity.

#### 3.3.1 Sponges

**Figure 31:**
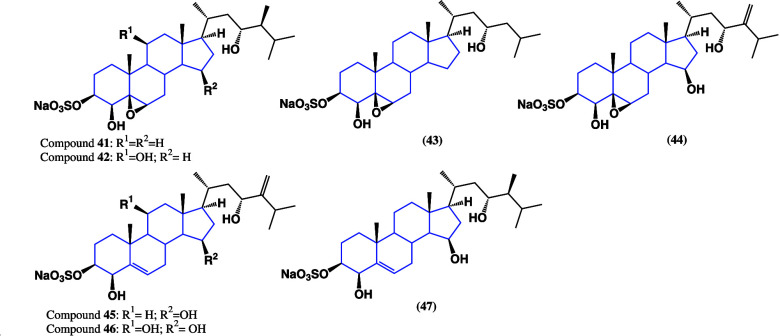
Structures of polyoxygenated steroids isolated from *Haliclona gracilis*.

Porifera (sponges) are one of the simpler and older organisms reported in the literature; they lack body symmetry, nervous and digestive systems, and true tissues. Their bodies have pores to allow water to pass through them to trap organic material and bacteria, and they have an important role in natural water treatment.^82^ Despite being simple organisms, they have complex metabolic processes allowing them to synthesize unique steroids, specially sulfated derivatives, and polyoxygenated compounds, with some of them proving effective against cancer progression.^83,84^ Seven new polyoxygenated steroids isolated from an ethanolic extract of the marine sponge *Haliclona gracilis* were reported.^85^ These compounds possess 3β-O-sulfonate-, 5β,6β-epoxy-, 5,6-dehydro-, and some other hydroxyl groups in diverse positions, e.g., 4β,23-dihydroxy substitution patterns as a common structural motif, as shown in Figure 31, denoting a higher degree of oxidation than those of natural origin, given the presence of hydroxyls in the rest of the structure as well as the formation of salts.

**Figure 32:**
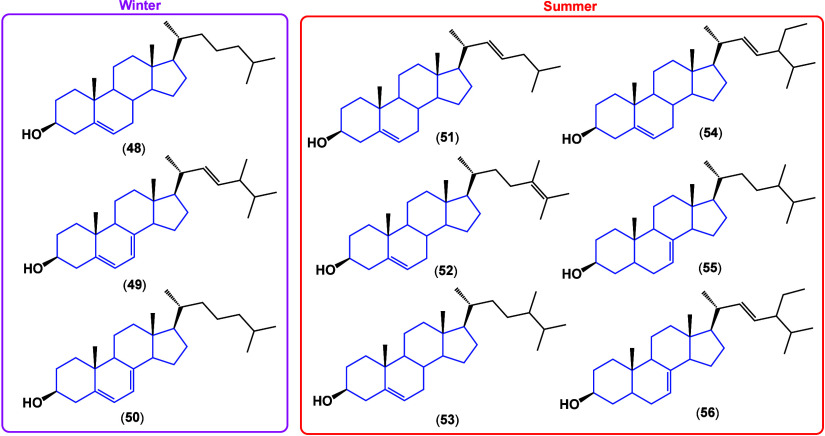
Structural difference between winter and summer extracts content.

The antitumor activity of compounds **41**, **42**, **45**, **46**, and **47** was evaluated against prostate cancer resistant to AR-targeted therapy cell line 22Rv1, which is an aggressive type of cancer with a low survival rate. All steroids exhibited moderate cytotoxic activity (emphasizing compound **47** with IC_50_ = 64.4 ± 14.9 μM) and were able to inhibit the expression of a prostate-specific antigen, indicating that inhibition may occur via PSA. On the other hand, the cytotoxic activity of several steroidal compounds isolated from methanol and dichloromethane extracts, from *Ircinia mutans* sponges, was evaluated.^86^ Interestingly, the steroid content varied depending on the season (summer or winter), presenting differences in the quantity and diversity of structures. Figure 32 shows the difference in steroids in winter and summer collections; however, the composition of these is variable depending on the temperature. Figure 32 shows those that were maintained between the extraction batches, highlighting that even compounds **48**, **49**, and **50** were detectable in summer although in very low proportions, demonstrating the viability of an exchange between structures resulting from the same metabolism of *Ircinia* mutans sponges, denoting its variability in production.

Winter collection steroids had the lowest IC_50_ values of 13.0 ± 0.9, 11.1 ± 1.7, and 1.1 ± 0.4 μg/mL against leukemia (MOLT-4), breast cancer (MCF-7), and colon cancer (HT-29), respectively, while those from the summer sample had IC_50_ 1.1 ± 0.2 μg/mL against MOLT-4. Cholestanic derivatives had great anticancer activity, at the level of *cis*-platinum, although it is not known whether it is by an antiproliferative or cytotoxic pathway.

**Figure 33:**
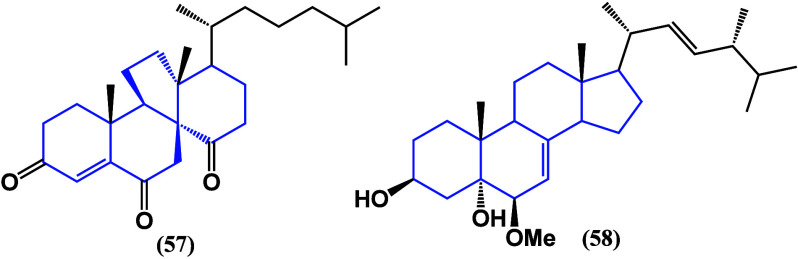
Structure of abeo-steroid **57** and ergostane derivative **58**.

A new steroid, 22,23-dihydro-24-nordankasterone A (**57**) (Figure 33) from the sea sponge *Luffariella variabilis* Western Central Pacific: Indonesia and Palau, was evaluated in the hormone-dependent MDA-MB-231 and K562 cancer cell lines^87^ and exhibited moderate cytotoxicity. It had IC_50_ values of 7.44 and 4.22 μM, respectively, retaining a high degree of oxidation in the structure as well as an anticancer activity, favored by the solidity of the derivatives.

**Figure 34:**
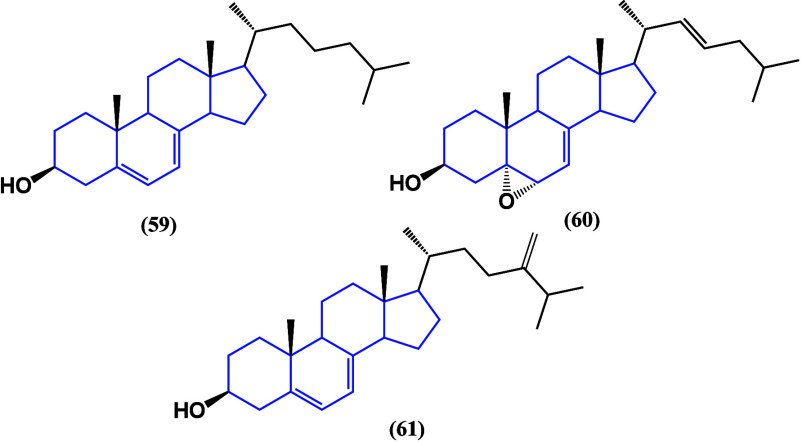
Structures of steroids **59**, **60**, and **61** isolated from *Phyllospongia sp*.

The ergostane derivative (22E)-6β-methoxyergosta-7,22-diene-3β,5α-diol (**58**) had moderate inhibitory effects on HL-60, K562, and BEL-7402 cancer cells with IC_50_ values ranging from 8.16 to 10.92 μg/mL.^88^ Three cholestane derivatives and one ergostane derivative isolated from *Phyllospongia sp*. from the South China Sea were tested for their cytotoxic activity against cancer cells. Cholest-5,7-diene-3β-ol (**59**), 5α,6α-epoxycholest-7,22-dien-3β-ol (**60**), and er-gosta-5,7,24(28)-trien-3β-ol (**61**) (Figure 34) were evaluated against MCF-7 human breast, HT-29 human colon, and HEP-2 human laryngeal cancer cells. Significant activity was observed against MCF-7, showing IC_50_ values of 8.8, 10.3, and 3.9 μM respectively.^89^ Despite a low selectivity with nontumorogenic cells, it gives rise to structural modifications.

#### 3.3.2 Sea Stars

**Figure 35:**
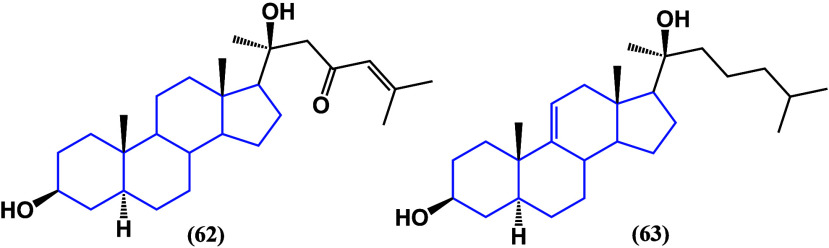
Novel steroidal **62** and **63** structures isolated from *Acanthaster planci*.

Some of the best-known marine animals are sea stars (incorrectly known as starfish). They are members of the phylum Echinodermata alongside sea urchins and sand dollars. These organisms play an important role in nature as active predators of clams, mussels, and other small forms of life. Echinoderms represent one of the most abundant groups of marine organisms with more than 7,000 members.^90^ Described as motionless organisms, their metabolism allows them to synthesize interesting secondary metabolites to protect them against adverse conditions at the bottom of the ocean and to defend themselves against predators. Within these interesting compounds, it is possible to find interesting bioactive molecules such as alkaloids, peptides, quinones, and steroids.^91,92^ A well-known broad spectrum of uses includes antibacterial^93,94^ and anticancer activity. Two new anticancer steroids were isolated from *Acanthaster planci*.^95^ Both compounds (Figure 35), (20*S*)-3β,20-dihydroxy-5α-cholest-24-en-23-one (**62**) and (20*S*)-5α-cholest-9(11)-en-3β,20-diol (63), had LC_50_ values like cisplatin (46 ± 1.1 μg/mL) with 49 ± 1.6 and 57.5 ± 1.5 μg/mL, respectively, in MCF-7 cell culture. Also, these steroids showed antibacterial activity against *Pseudomonas aeruginosa* and antidiabetic action, proving their pharmacological potential.

**Figure 36:**
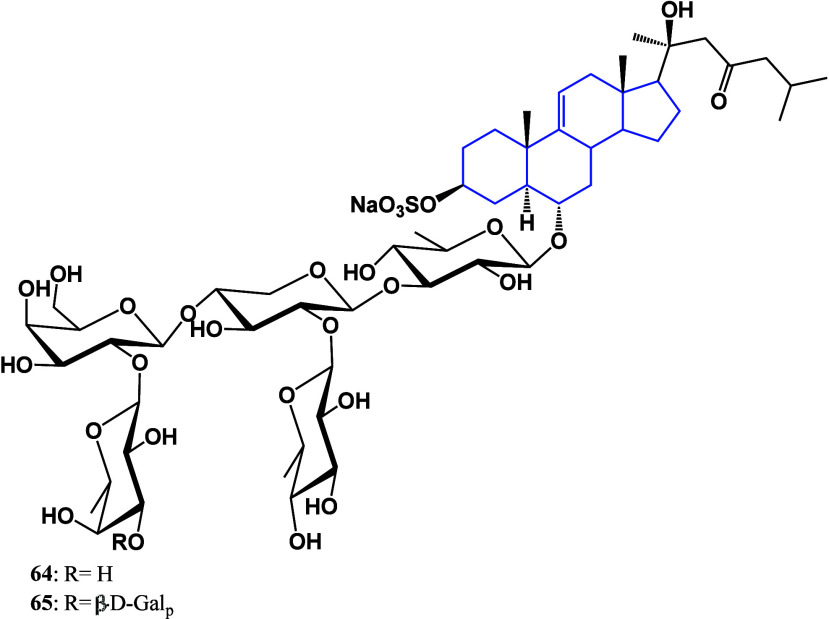
Anticancer steroids isolated from *Asterias microdiscus*.

Due to their broad distribution in oceans, sea star-derived steroids change abruptly according to their geological position, for example, two new structures from the Arctic sea star *Asterias microdiscus*: the sulfated polyhydroxysteroid microdiscusol G (a side chain never reported before in sea stars) and the polyhydroxysteroid bioside, microdiscusoside A. Their elucidation was carried out using 1D and 2D NMR spectroscopy.^96^ Saponins **64** and **65** shown in Figure 36 exhibited cytotoxic effects against HT-29, MDA-MB-231, THP-1, and Raji and also suppressed cell proliferation and colony formation of cancer cells HT-29 and MDA-MB-231 in nontoxic concentrations. Recovering the high potential of steroidal saponins in cancer cell lines, although with the main disadvantage of the low concentrations in which they are obtained from their origin, returning to the effect of the combination with carbohydrates increases the activity. Particularly by presenting molecules with a higher degree of oxidation, the cytotoxic effect in healthy lines decreased, giving rise to structural optimization.

**Figure 37:**
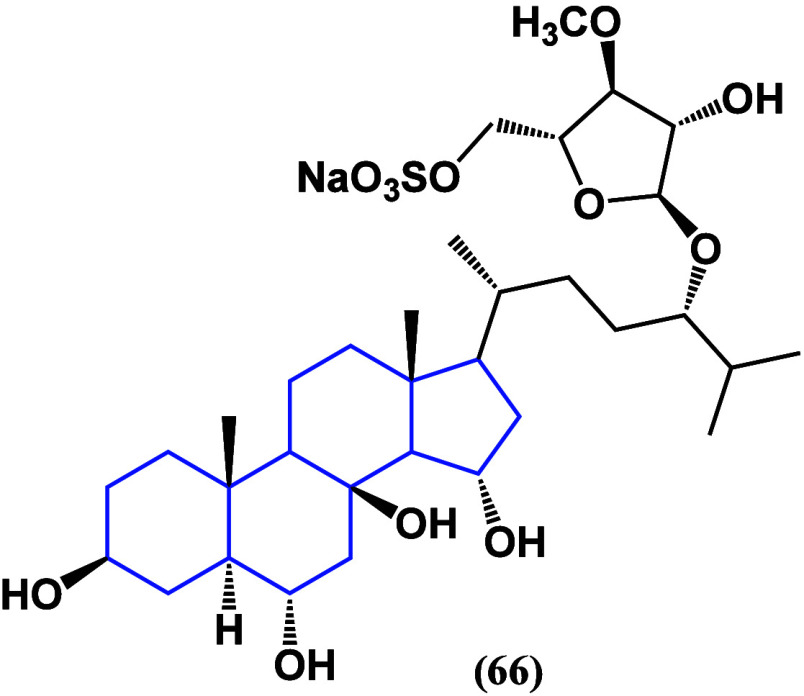
Structure of asterosaponin P1 (**66**).

In many types of cancer, radiotherapy is a first-row option to eliminate localized tumors and has been proven to be effective in many patients. Nevertheless, there are tumors resistant to radiotherapy, showing a high mortality rate. It has been reported that asterosaponin P1 (**66**) (Figure 37) has remarkable radio-sensitizing activity in HT-29 cells and can induce apoptosis by caspase activation.^97^

#### 3.3.3 Algal Steroids

**Figure 38:**
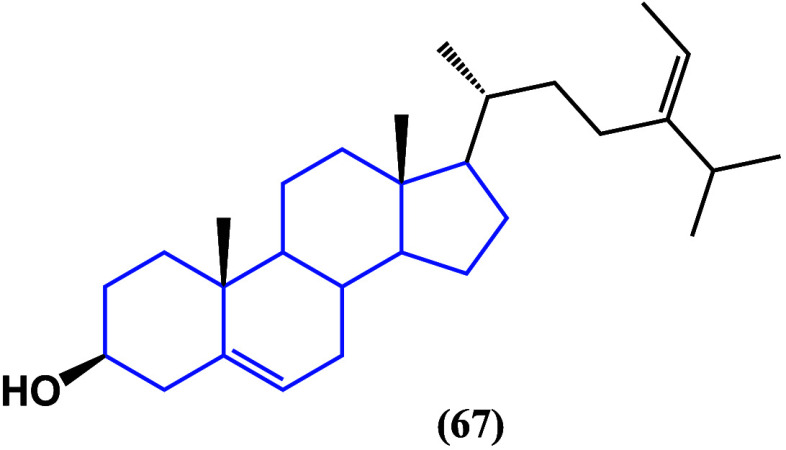
Structure of fucosterol (**67**).

Fucosterol (**67**) (Figure 38) is a steroid isolated from brown algae that has been proven to have important anticancer bioactivity. Using molecular docking, it was proposed that the molecular mechanism of the anticancer activity against non-small-cell lung cancer may occur via MAPK1, EGFR, GRB2, IGF2, MAPK8, and SRC, which could explain the regulation of the apoptosis process.^98^

**Figure 39:**
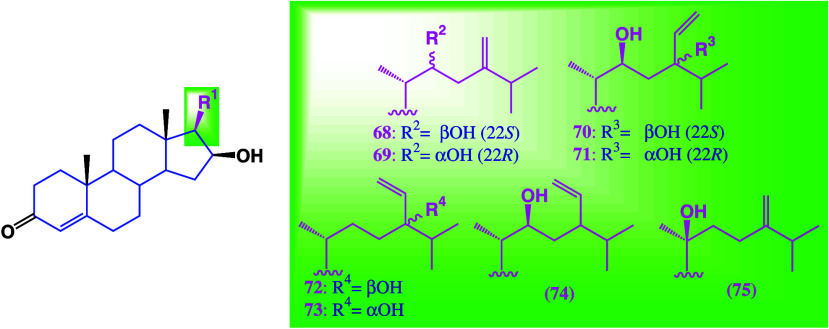
Structures of compounds isolated from *Cystophora xiphocarpa*.

Fucosterol has activity against ovarian cancer, inhibiting its proliferation and cell cycle by activating caspase-3, caspase-9, and cytochrome C due to mitochondrial dysfunction.^99^ Also, fucosterol inhibited signal transduction pathways including P13K and MAPK. A series of steroids from the brown alga *Cystophora xiphocarpa*, compounds **68**, **69**, **72**, **73**, **74**, and **75** (Figure 39),^100^ were tested against 12 cancer cell lines: HT29, SW480, MCF-7, A2780, H460, A431, Dul45, BE2-C, SJ-G2, SMA, U87, and MIA. Compound **75** had the best cytotoxic activity, with GI50 8.7 ± 0.7 μM against HT29, GI_50_ 5.6 ± 0.8 μM against breast cancer line MCF-7, and GI50 4.5 ± 0.2 μM against ovarian cancer cell line A2780.

**Figure 40:**
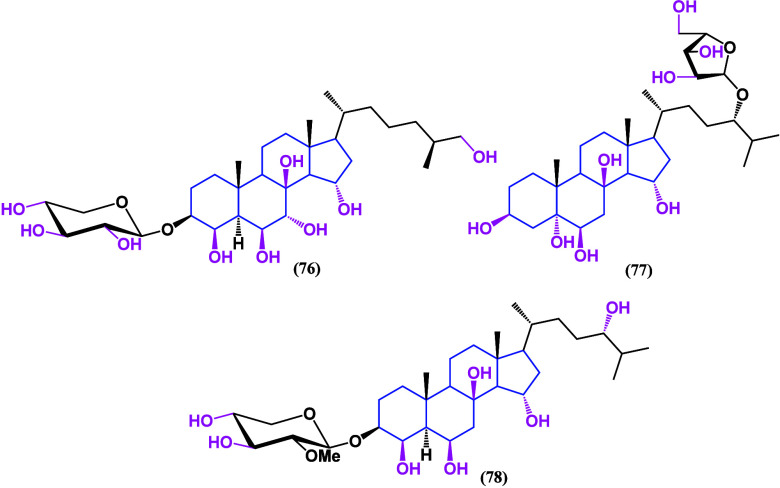
Steroids isolated from sea star *Protoreaster lincki*.

Interestingly, it has been seen that the combined action of sulfated laminaran from two different marine sources and steroid fractions has an effect against cancer. The synergic action in 3D culture cancer cell models of polyoxygenated steroidal glycosides (Figure 40), isolated from the brown alga *Alaria angusta* and the starfish *Protoreaster lincki*, was established. All steroids inhibited cell proliferation and invasion in HCT116 3D culture, but polyoxygenated steroids from brown algae in combination with **75** induced apoptosis through the inactivation of protein kinase B.^101^

The marine origin for the isolation of steroids mainly gives compounds with a higher degree of oxidation. This is associated with the presence of hydroxyl and ketone groups, with high biological activity against cancer cell lines, regardless of whether it is hormone- or non-hormone-dependent. In addition to the combination with carbohydrates, steroidal saponins are the targets to obtain and modify.

## 4 Synthetic Steroids

As seen above, some natural steroids have intrinsic anticancer activity; however, it has been shown that chemical modifications of steroids are effective in improving biological activity and increasing selectivity against cancer cells while avoiding damaging healthy cells. In addition to the low bioavailability of these from natural sources, the main focus is obtaining these, in addition to structural modifications to increase their activity. After discovering the high biological potential of steroid derivatives in order to obtain the previously isolated structures or even generate modifications in order to increase the biological activity or selectivity, the synthesis or semisynthesis of steroid derivatives is necessary.

### 4.1 Steroidal Oximes

**Scheme 1:**
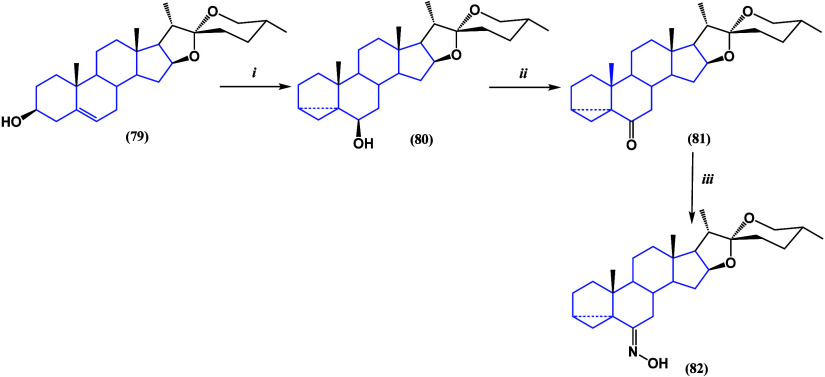
(i) Steroidal Oxime Obtained; (ii) TsOH, py; KOAc, aq.; and (iii) NBS; NH_2_OH·HCl

The potential activity of steroidal oximes such as compound **82** (Scheme 1) was searched.^102^ This derivative showed selectivity toward the triple-negative MDA-MB-231 cell line with remarkable biological activity at concentrations below 10 μM. The raw material for their synthesis was diosgenin (**79**), a compound that has been proven to be effective against breast cancer, but its chemical modification to produce oxime compound **78** enhanced its biological activity.

**Scheme 2:**
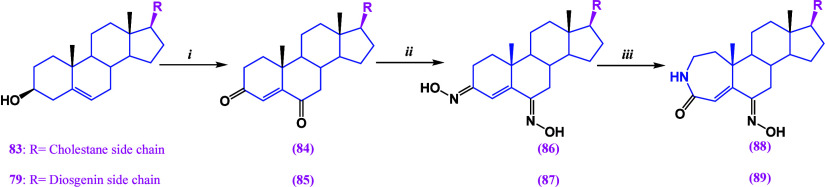
(i) Jones Reagent; (ii) NH_2_OH·HCl, NaOAc; and (iii) SOCl_2_

**Scheme 3:**
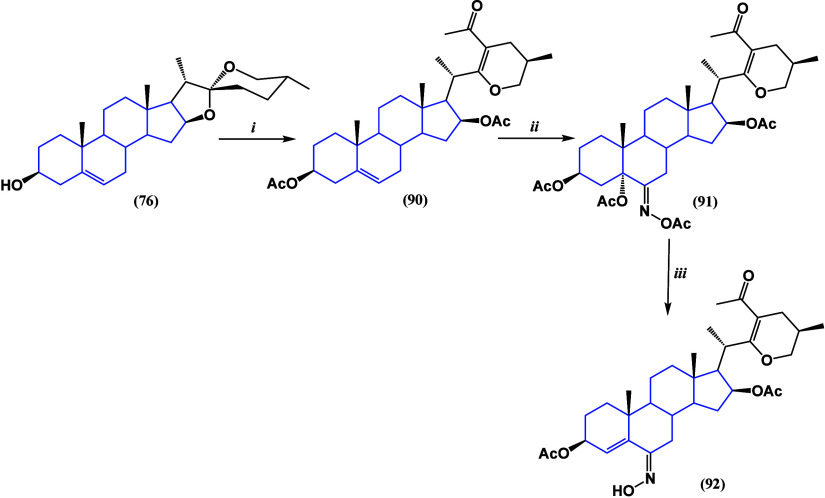
(i) Ac_2_O, Et_2_O·BF_3_; (ii) NaNO_2_, Et_2_O·BF_3_, Ac_2_O/AcOH; and (iii) Na_2_CO_3_

Starting from diosgenin, a series of derivatives were synthesized (Schemes 2 and 3)^103^ and evaluated against breast cancer cell line MCF-7. The most potent derivatives were identified as compounds **86**, **89**, and **92**, displaying remarkable activity with IC_50_ values ranging from 7.9 to 9.5 μM. Notably, these compounds exhibited excellent selectivity with IC_50_ values exceeding 100 μM against the nontumor cell line.

**Scheme 4:**
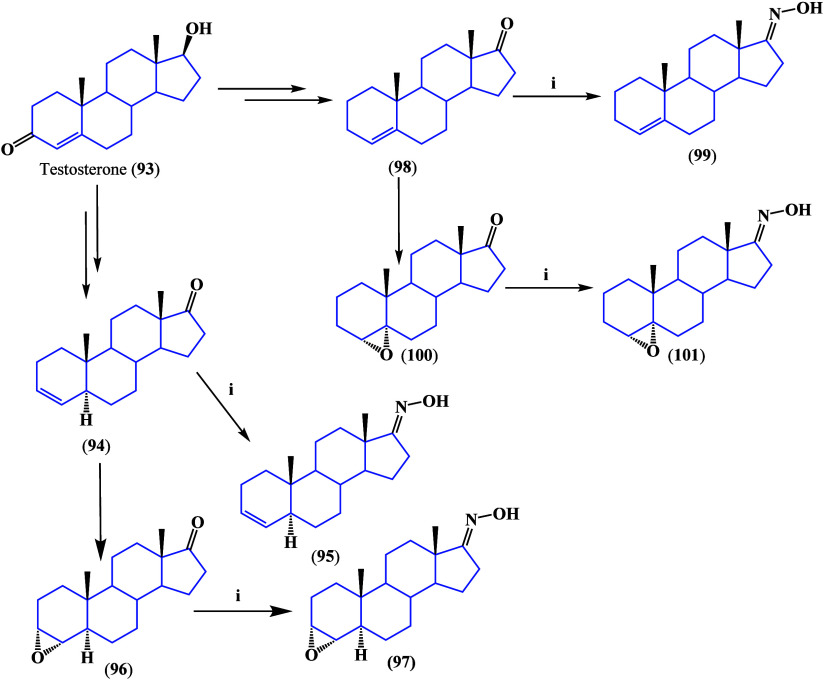
Testosterone Oxime Derivates: (i) NH_2_OH·HCl, CH_3_COONa·3H_2_O, Methanol, 40 °C

It has also been reported that derivatives of testosterone are effective against several cancer panels. Four steroidal oximes were synthesized as shown in Scheme 4. Only compounds with double bonds at positions C-3 and C-4 presented good antiproliferative activity against all types of studied cancer lines. These structures were able to induce apoptosis and cell cycle arrest and increased the production of ROS in prostate cell line PC3 and colon adenocarcinoma cell line WiDr, showing higher cytotoxicity activity in these cell lines.^104^

**Scheme 5:**
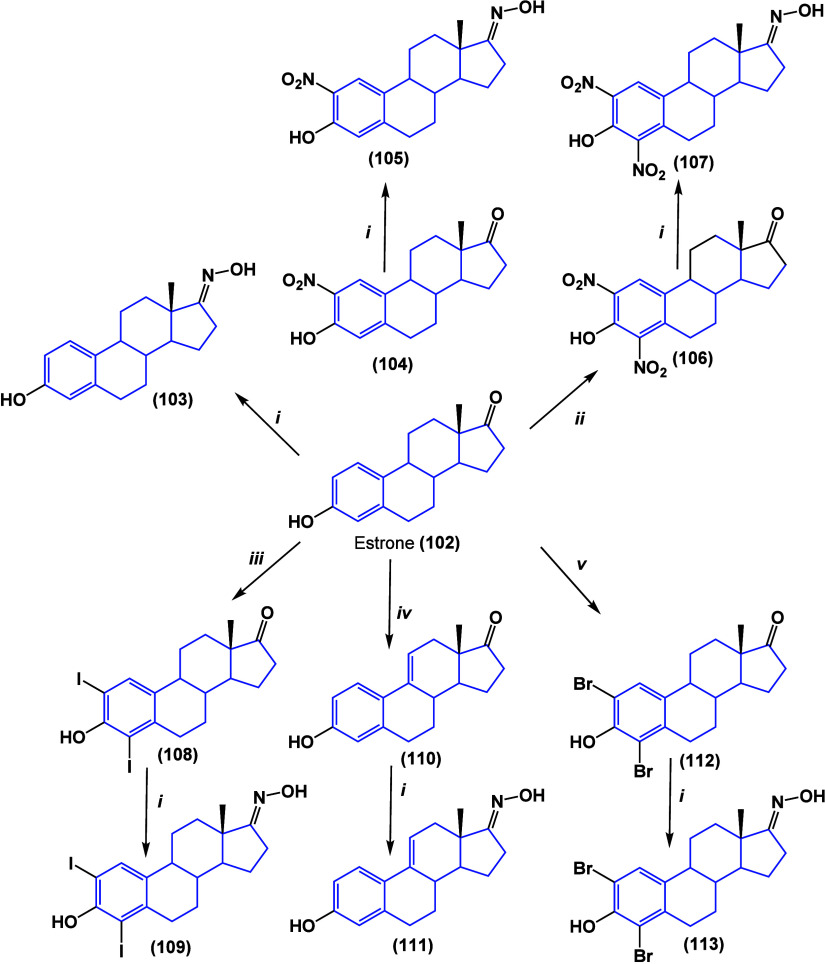
(i) NH_2_OH HCl, NaOH; (ii) HNO_3_, AcOH; (iii) I_2_; (iv) DDQ; and (v) N-Bromosuccinimide

On the other hand, assays with estrone were also performed, and the cytotoxic activity for estrone oximes (Scheme 5) was evaluated against six cell lines. Oximes reduced cancer cell populations, and all compounds presented activity against MCF-7 and HepaRG. Oxime compound **111** bearing a double bond at position 9 showed improved results against the LNCaP cell line and promoted the condensation and fragmentation of DNA, indicating apoptosis as the cell death mechanism. However, this compound promoted the proliferation of T47-D cells.^105^

### 4.2 Bile Acid Derivatives

Due to the high bioactivity of bile acids in cancer cells and their regulation role in disease incidence and progression, bile acids act as scaffolds to synthesize compounds with enhanced anticancer activity.

**Scheme 6:**
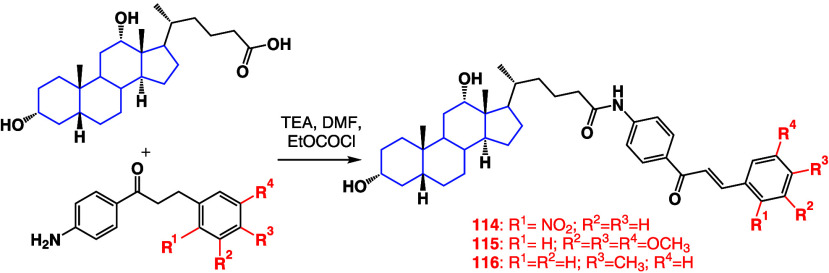
Synthesis of Chalcone-Deoxycholic Acid Conjugates **114** and **116**

A series of deoxycholic acid-chalcone conjugates^106^ (Scheme 6) were synthesized, and it was found that chalcone moieties borne to 2-nitrophenyl and 3,4,5-trimethoxyohenyl groups (compounds **114** and **115**) exhibited the best bioactivity against cervical cancer cell line SiHa, with IC_50_ values of 0.51 and 0.84 μM. On the other hand, compounds **114** and **116** had the best activity against lung cancer cell line A549 with IC_50_ values of 0.25 and 1.71 μM, respectively.

**Figure 41:**
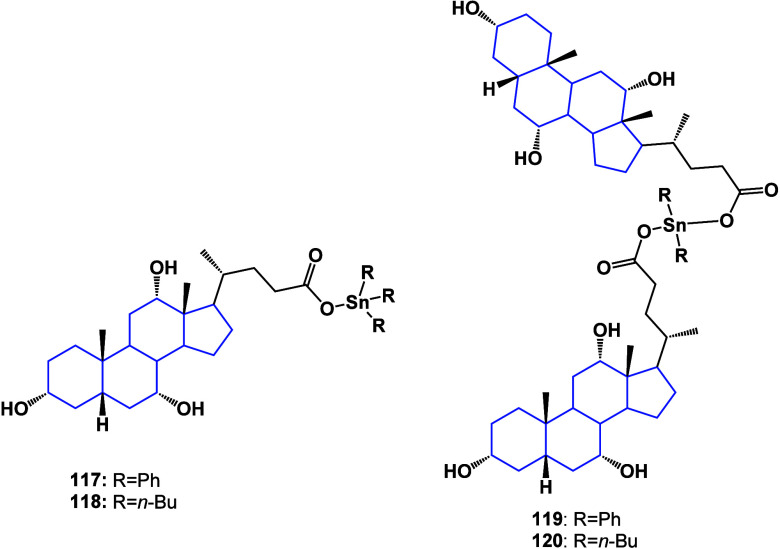
Organotin derivatives of cholic acid.

Cholic acid, which is an important bile acid present in bile, has also been chemically modified to increase its activity against breast cancer. The synthesis and evaluation of organotin(IV) derivatives of cholic acid^107^ are shown (Figure 41). Compounds **117**–**120** inhibit cell proliferation in both MCF-7 and MDA-MB-231 cell lines; derivatives **117** and **118** showed selectivity toward MCF-7.

Cholic acid has also been used as a scaffold to synthesize drug carriers to increase the aqueous solubility of sorafenib, an important drug against many types of cancers. In this sense, an increased solubility was observed when using the dual chitosan conjugate with cholic acid and galactose, enhancing the aqueous solubility of sorafenib by about 1117-fold (from 1.7 to 1900 μg/mL).^108^

**Figure 42:**
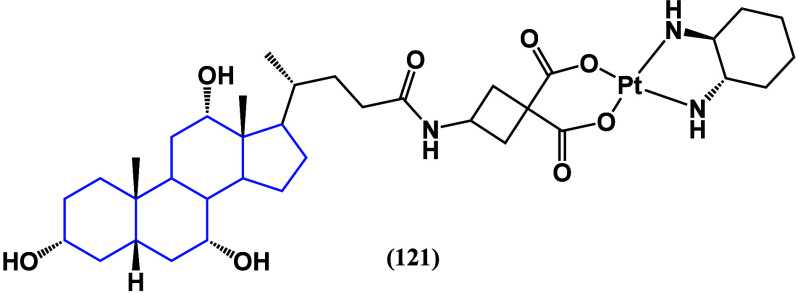
Structure of LLC-202 (**121**).

Recently, cholic acid has been reported as a scaffold to synthesize prodrugs against liver cancer. *cis*-Platin was linked to bile acid to target liver cells by binding with FXR, promoting better cell uptake and pharmacodynamics. Molecule LLC-202 (**121**, Figure 42) exhibits higher *in vitro* anticancer activity and higher efficacy compared to oxaliplatin in treating primary hepatocellular carcinoma.^109^

**Figure 43:**
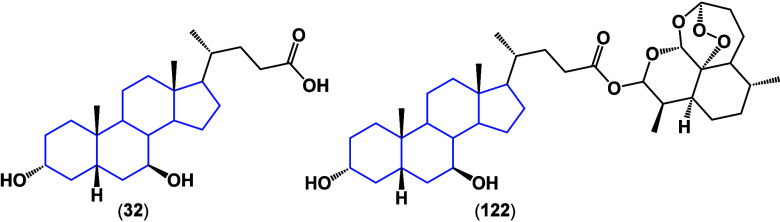
Structures of ursodeoxycholic acid (**32**) and its conjugate with dihydroartemisin (**122**).

Conjugate **122** of ursodeoxycholic acid (**32**) with dihydroartemisin (Figure 43) was tested against hepatocellular carcinoma cancer cell line Huh-7, obtaining an IC_50_ of 2.16 μM, inducing depolarization of the mitochondrial membrane.^110^

### 4.3 22-Oxocholestane Derivatives

**Scheme 7:**
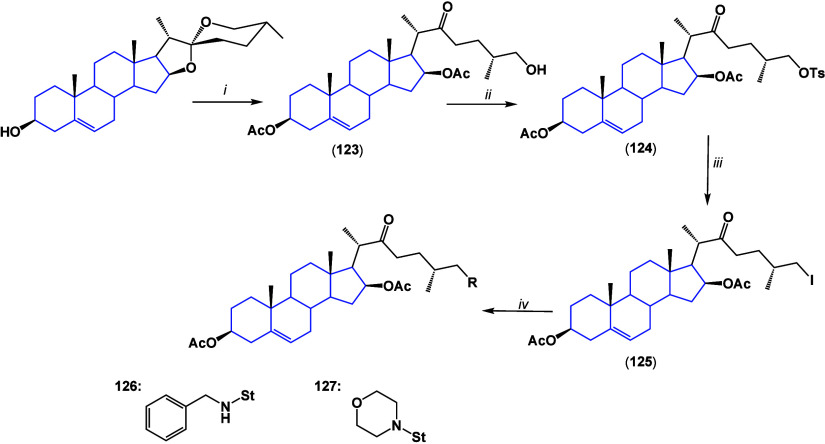
(i) Ac_2_O, Et_2_O·BF_3_/Et_3_N; HCl aq.; (ii) pTsCl/Py/DMAP; (iii) NaI; and (iv) Amine/CH_3_CN

22-Oxocholestane structures have shown promising anticancer activity due to their structural similarity to cholesterol and bearing the carbonyl pharmacophore at C-22. Some 26-amino-22-oxocholestanes were synthesized^111^ and evaluated. The cytotoxic activity of **126** in SiHa cancer cells exhibited good activity with a half-maximum cytotoxicity concentration (CC_50_) of 6.63 μM, while **127** presented moderate cytotoxic activity on SiHa and MCF-7 with CC_50_ values of 15.34 and 16.33 μM, respectively (Scheme 7).

**Figure 44:**
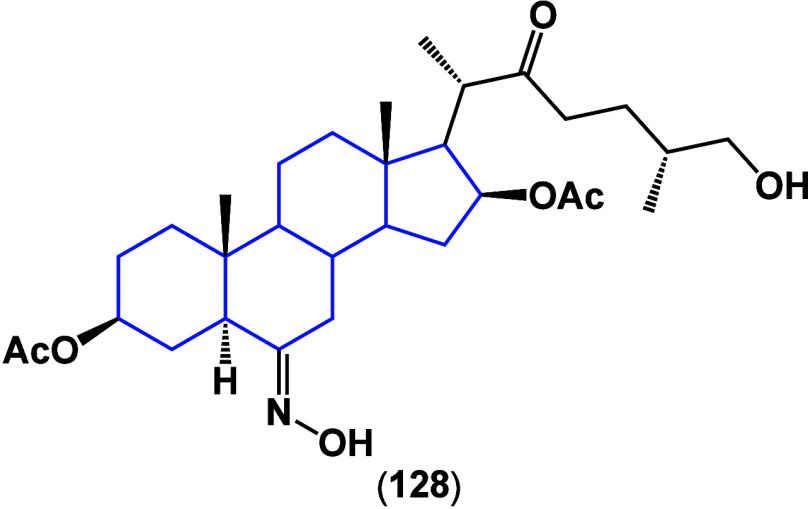
Structure of compound **128**.

The activity of compound **128** (Figure 44) on the MCF-7 cell line was reported,^112^ signaling to have an IC_50_ of 9.3 μM while MDA-MB-231 did not show inhibitory activity beyond 100 μM.

**Figure 45:**
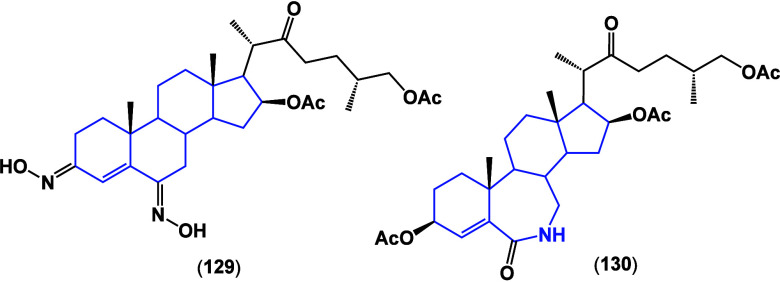
Structures of compounds **129** and **130**.

A series of compounds derived from diosgenin and cholesterol were synthesized; compounds **126** and **127** (Figure 45) were evaluated against the MCF-7 cell line, finding IC_50_ values of 30.5 and 24.2 μM, respectively.^113^ Confirming the need for oxygenated groups in combination with nitrogen within the structure increases the antiproliferative biological activity within steroidal structures.

## 5 Conclusions

Steroids have great potential as anticancer agents, mainly through the antiproliferative pathway, cell cycle arrest pathways, and cell death pathway, highlighting the induction of apoptosis. However, they are not always selective against healthy cells. In the last 4 years, more than 100 molecules have been designed and evaluated in different types of cancer, from hormone-dependent breast, cervical, and prostate cancers to non-hormone-dependent cancers such as triple-negative breast, lung, and colon cancers, among others. We highlighted two main modifications, one focused on the inclusion of carbohydrates, forming saponins, although with low selectivity against healthy cells, and the second increasing the oxidation state, mainly via oxygen and nitrogen, within both the steroid skeleton and substituents. In particular, the combination of carbohydrates in the presence of nitrogen gave rise to compounds selective against healthy cells. This gives rise to many structural modifications to improve biological activity, generating criteria for the inclusion of these heteroatoms as well as bioconjugation with carbohydrates, paving the way for future research in the field of anticancer steroids.
